# Comparison of Preoperative Alpha-Blocker Therapy Versus Placebo Before Ureterorenoscopy and Lithotripsy for Distal Ureteral Stones

**DOI:** 10.7759/cureus.95119

**Published:** 2025-10-22

**Authors:** Muhammad Sanan, Zabish Mehmood, Muhammad Asad Abdullah, Hamna Fayyaz Fayyaz, Momina Asif, Hira A Bumbia, Shehroze Rauf Shakoori

**Affiliations:** 1 Urology, Bahawal Victoria Hospital, Quaid-e-Azam Medical College, Bahawalpur, PAK; 2 Urology, Sir Ganga Ram Hospital, Lahore, PAK; 3 Surgery, Central Park Medical College, Lahore, PAK; 4 General Medicine, Amna Inayat Medical College, Lahore, PAK; 5 Urology, Dr. Ziauddin Hospital, Karachi, PAK

**Keywords:** complications, outcomes, patients, preoperative alpha blocker, ureterorenoscopy

## Abstract

Introduction

Ureteric colic caused by distal ureteral stones is a common clinical condition that often requires intervention. The American Urological Association recommends the use of alpha-blockers for stones measuring ≤10 mm, as these drugs help relax the ureteric smooth muscle and facilitate stone passage.

Objectives

The objective of this study was to compare the efficacy of preoperative alpha-blocker therapy versus placebo in improving the success rate and reducing complications during ureterorenoscopy (URS) for distal ureteral stones.

Methodology

This comparative observational study was conducted in the Department of Urology and Renal Transplantation, Bahawal Victoria Hospital, Bahawalpur, Pakistan, from March 2022 to September 2022. A total of 88 patients aged 25-65 years with distal ureteric stones of more than one month in duration were included. Patients with a history of ureteric surgery, high-grade hydronephrosis, ureteric pathology, pregnancy, spontaneous stone passage, solitary kidney, bilateral ureteric stones, prior medical expulsive therapy, or long-term use of α-adrenergic receptor blockers for benign prostatic hyperplasia were excluded. In Group A, URS and lithotripsy were performed after two weeks of alpha-blocker therapy (silodosin 8 mg once daily), while in Group B, URS and lithotripsy were performed after two weeks without alpha-blocker therapy (placebo group). Patients who expelled stones before the procedure were excluded. Effectiveness was evaluated after one week.

Results

The mean age of patients in Group A was 38.23 ± 8.26 years, and in Group B it was 37.46 ± 9.97 years. The majority of the patients, 65 (73.86%), were between 25 and 45 years of age. Of the 88 patients, 53 (60.23%) were male and 35 (39.77%) were female, with a male-to-female ratio of 1.8:1. In the present study, the efficacy, defined as successful URS without ureteral dilatation and absence of complications, of URS lithotripsy with alpha-blocker therapy was 81.82%, compared with 54.55% in URS lithotripsy without alpha-blocker therapy (p = 0.006), which was statistically significant.

Conclusions

Preoperative alpha-blocker therapy significantly improves the success of URS and reduces complications in patients with distal ureteral stones. The use of alpha-blockers is recommended to enhance procedural outcomes and minimize the risk of complications during URS and lithotripsy. Further studies with larger sample sizes and longer follow-up periods are warranted to confirm these findings.

## Introduction

Ureteric colic is a type of pain caused by an obstruction in the ureter. It can be severe and requires prompt management [1]. The pain usually begins in the loin (flank) and radiates down to the groin and anterior abdomen. It may also extend to the labia in females or the testicle in males. Ureteric colic occurs when a stone moves down the ureter, leading to obstruction. Patients presenting to emergency departments or outpatient urology clinics commonly report acute flank pain due to urolithiasis [2]. Obstructing ureteral stones are considered “impacted into the ureteric wall” if they remain in the same position for a defined period (more than one month) [1].

The prevalence of urolithiasis in South and Southeast Asia, including Pakistan, is approximately 16%, and it is significantly higher in regions with elevated temperatures and high sun exposure [2]. Various imaging techniques are frequently used in the diagnosis of urolithiasis, and these methods continue to improve over time in identifying ureteric stones. Intravenous urography has long been considered the preferred radiographic technique for evaluating the ureter, bladder, and intrarenal collecting system [3].

Urolithiasis is a growing global health concern, with type 2 diabetes mellitus, obesity, and metabolic syndrome identified as major contributing factors [4]. Ureteric stones, which affect approximately 12% of men and 6% of women during their lifetime, are among the most common causes of emergency room visits [5]. Nearly 70% of these stones are distal ureteric stones, which are typically symptomatic [6].

Management options for ureteric stones include medical expulsive therapy (MET), shockwave lithotripsy, ureteroscopy, percutaneous nephrolithotomy, open or laparoscopic stone surgery, and observation, depending on the clinical scenario [7,8]. The American Urological Association recommends the use of alpha-blockers, a practice supported by several meta-analyses, particularly for stones measuring ≤10 mm [9]. The vesicoureteric junction contains the highest concentration of ureteric alpha receptors, which regulate peristalsis through segmental contractions. Alpha-blockers inhibit this activity by relaxing ureteric smooth muscle, dilating the ureter and ureteric orifice, and reducing the frequency and amplitude of peristalsis. Hydration, as part of MET, further assists in stone passage [10].

The objective of this study was to compare the efficacy of preoperative alpha-blocker therapy versus placebo in improving the success rate and reducing complications during ureterorenoscopy (URS) for distal ureteral stones.

## Materials and methods

This comparative, non-randomized observational study was conducted on 88 patients admitted to the Department of Urology and Renal Transplantation, Bahawal Victoria Hospital, Bahawalpur, Pakistan, from March 2022 to September 2022. Ethical approval was obtained from the hospital’s ethics committee for the use of patient medical data and publication of the study results. Patient confidentiality was strictly maintained throughout the study.

The study compared outcomes between two groups: patients who received preoperative alpha-blocker therapy and those who did not receive any preoperative medication, with the aim of assessing differences in procedural success and complication rates. A consecutive sampling technique was used based on specific inclusion and exclusion criteria.

Patients were included if they had a distal ureteric stone, as per the operational definition, with a duration of more than one month, a stone size between 8 and 20 mm, and were between 25 and 65 years of age of either gender. Patients were excluded if they had a history of MET, a solitary kidney, bilateral ureteric stones, previous ureteric surgery, high-grade hydronephrosis, ureteric pathology, pregnancy, a history of stone passage, or long-term use of α-adrenergic receptor blockers for benign prostatic hyperplasia.

Data collection

A total of 88 patients who attended the outpatient department of the Department of Urology and Renal Transplantation, Bahawal Victoria Hospital, Bahawalpur, and met the inclusion criteria were enrolled in the study. Informed consent was obtained from each participant. The selected patients were divided equally into two groups, A and B, with 44 patients in each group. The sample size was calculated using the WHO sample size calculator for two proportions, with a 5% level of significance and 80% study power, taking the efficacy (defined as successful URS without complications) of preoperative alpha-blocker therapy as 96.8% and that of the placebo group as 80.0% [11].

In Group A, URS and lithotripsy were performed after two weeks of alpha-blocker therapy (silodosin 8 mg once daily), while in Group B, URS and lithotripsy were performed after two weeks without alpha-blocker administration (placebo group). All procedures were carried out by a single surgeon. Patients who expelled stones before the procedure were excluded.

Efficacy was defined as successful URS without ureteral dilatation and the absence of complications. Complications were classified as follows: (1) septicemia, considered present if the patient exhibited at least three of the following: fever (>38 °C), tachypnea (>20 breaths/min), or tachycardia (>90 beats/min); (2) bleeding, identified by the presence of postoperative hematuria; and (3) UTI, defined by pyuria (≥4 WBCs/HPF in males and ≥7 WBCs/HPF in females) and bacteriuria (≥10⁵ CFU/mL). All complications were assessed after one week.

The following variables were recorded for each patient: age, gender, BMI (>30 considered obese and ≤30 non-obese), stone size, side effects, and efficacy (yes/no) [12].

Data analysis

Statistical analysis was performed using IBM SPSS Statistics for Windows, Version 25.0 (Released 2017; IBM Corp., Armonk, NY, USA). The Shapiro-Wilk test was applied to assess the normality of continuous data. Continuous variables such as age, disease duration, and stone size were expressed as mean ± SD or median (IQR), as appropriate. Categorical variables, including gender, affected side, complications (septicemia, bleeding, and UTI), ureteral dilatation, and efficacy (yes/no), were presented as frequencies and percentages.

The chi-square test or Fisher’s exact test, as appropriate, was used to evaluate the differences in efficacy between the two study groups. A p-value of less than 0.05 was considered statistically significant. Stratification was performed for age, gender, duration of illness, affected side, and stone size to control for potential effect modifiers.

## Results

The age of patients in the present study ranged from 25 to 65 years, with a mean age of 37.23 ± 9.69 years. The mean age of patients in Group A was slightly higher compared with that of Group B. The majority of patients, 65 (73.86%), were between 25 and 45 years of age. Regarding gender distribution, the proportion of male patients was higher than that of female patients, with a male-to-female ratio of 1.8:1. The mean stone size was 11.62 ± 1.62 mm, and the mean disease duration was 5.23 ± 2.69 months (Table 1).

**Table 1:** Distribution of patients based on age, gender, stone size, duration of stay, side effects, and efficacy Values are expressed as frequency (percentage).

Factor	Group A (N = 44)	Group B (N = 44)	Total (N = 88)	Percentage (%)
Age (years)				
25-45	32	33	65	73.86
46-65	12	11	23	26.14
Gender				
Male	28	25	53	60.23
Female	16	19	35	39.77
Stone size (mm)				
8-15	36	35	71	80.68
16-20	8	9	17	19.32
Duration of disease (months)				
≤6 months	32	33	65	73.86
>6 months	12	11	23	26.14
Affected side				
Right	24	22	46	52.27
Left	20	22	42	47.73
Side effects reported				
Septicemia	3 (6.82%)	6 (13.64%)	9	10.23
Bleeding	2 (4.55%)	4 (9.09%)	6	6.82
UTI	4 (9.09%)	5 (11.36%)	9	10.23
Efficacy				
Yes	36 (81.82%)	24 (54.55%)	60	68.18
No	8 (18.18%)	20 (45.45%)	28	31.81

In the present study, the efficacy, defined as successful URS without ureteral dilatation and the absence of complications, was 81.82% in the group that received alpha-blocker therapy, compared with 54.55% in the group that did not receive alpha-blockers. The difference was statistically significant (p = 0.006). The complication rates observed in both groups are summarized in Table 2.

**Table 2:** Complication rates in Group A and Group B Septicemia was defined as the presence of three or more systemic inflammatory signs (fever >38°C, respiratory rate >20/min, and heart rate >90/min). Postoperative bleeding referred to hematuria observed after the procedure, and UTI was defined as pyuria (WBC >4/HPF in males and >7/HPF in females) with bacteriuria (≥10⁵ CFU/mL). Comparisons between groups were performed using the chi-square test or Fisher’s exact test, as appropriate. A p-value of <0.05 was considered statistically significant.

Complications		Group A	Group B	p-Value
		N (%)	N (%)	
Septicemia	Yes	3 (6.82%)	6 (13.64%)	0.291
	No	41 (93.18%)	38 (86.36%)	
Bleeding	Yes	2 (4.55%)	4 (9.09%)	0.398
	No	42 (95.45%)	40 (90.91%)	
UTI	Yes	4 (9.09%)	5 (11.36%)	0.725
	No	40 (90.91%)	39 (88.64%)	

Stratification of efficacy with respect to age, gender, duration of disease, stone size, and affected side is presented in Table 3.

**Table 3:** Stratification of efficacy with respect to age, gender, duration of disease, stone size, and affected side Comparisons between groups were performed using the chi-square test. A p-value of <0.05 was considered statistically significant.

Variable		Group A (N = 44)		Group B (N = 44)		p-Value
		Efficacy		Efficacy		
		Yes	No	Yes	No	
Age (years)	25-45	24 (75.0%)	8 (25.0%)	15 (45.45%)	18 (54.55%)	0.015
	46-65	12 (100.0%)	0 (0.0%)	9 (81.82%)	2 (18.18%)	0.122
Gender	Male	24 (85.71%)	4 (14.29%)	20 (80.0%)	5 (20.0%)	0.58
	Female	12 (75.0%)	4 (25.0%)	4 (21.05%)	15 (78.95%)	0.001
Size of stone (mm)	8-15	29 (80.56%)	7 (19.44%)	16 (45.71%)	19 (54.29%)	0.002
	16-20	7 (87.50%)	1 (12.50%)	8 (88.89%)	1 (11.11%)	0.929
Duration (month)	≤6	24 (75.0%)	8 (25.0%)	15 (45.45%)	18 (54.55%)	0.015
	>6	12 (100.0%)	0 (0.0%)	9 (81.82%)	2 (18.18%)	0.122
Affected side	Right	24 (100.0%)	0 (0.0%)	18 (81.82%)	4 (18.18%)	0.029
	Left	12 (60.0%)	8 (40.0%)	6 (27.27%)	16 (72.73%)	0.032

## Discussion

Much effort has been made to improve the management of ureteric calculi through URS, with the introduction of numerous instruments and procedural modifications [11]. Advancements in URS techniques, such as the “chip at the tip” design, improved extraction baskets, and ureteroscopes equipped with distal digital cameras, have undoubtedly enhanced stone clearance rates. Consequently, URS is expected to become increasingly safer and more efficient in the future. Likewise, the spontaneous passage rate of calculi has significantly improved since the introduction of alpha and calcium channel blockers prior to URS [12]. The preoperative use of these medications, particularly alpha-blockers, has also facilitated easier URS procedures.

The present study aimed to compare the efficacy of preoperative alpha-blocker therapy and placebo before URS for ureteral stones. In this study, the efficacy of URS lithotripsy with an alpha-blocker (defined as successful URS without ureteral dilatation and absence of complications) was 81.82%, whereas the efficacy without an alpha-blocker was 54.55%, with a statistically significant p-value of 0.006. In a previous trial, the efficacy of preoperative alpha-blocker therapy was reported as 96.8%, compared with 80.0% in the placebo group [13]. The placebo group also had a significantly higher complication rate than the alpha-blocker group (20% vs. 6.4%; p = 0.036). Another study found that silodosin was 70.6% effective (no ureteral dilatation required) compared with 32.4% in the placebo group [14].

Data from nine studies involving more than 700 patients indicated that individuals receiving alpha-blockers had a 54% higher likelihood of spontaneous stone passage than those not receiving the treatment, according to one of Hollingsworth’s early meta-analyses [15]. However, the large, double-blind, multicenter Spontaneous Urinary Stone Passage Enabled by Drugs (SUSPEND) trial found no benefit of active therapy. With an adjusted risk difference of 1.3% (95% CI: 5.7-8.3; p = 0.73), 80% of patients in the placebo group and 81% in the tamsulosin group did not require further intervention [16].

An analytical study by Tan et al., which included a pooled analysis of trials conducted between January 1980 and June 2019, evaluated the role of alpha-blockers as adjuvant therapy before URS for ureteric calculi [4]. The findings, consistent with those of the present study, demonstrated that preoperative alpha-blocker use was associated with a significantly lower risk of complications, higher successful access rates to the stone, a higher stone-free rate at four weeks, and a reduced need for balloon dilatation. However, this analysis showed no significant difference in operative time between groups, which contrasts with our findings.

The results of a 2018 trial by Mohey et al., which used 8 mg of silodosin as preoperative alpha-blocker therapy, were also similar to our results [10]. The silodosin group experienced easier ureteroscope advancement, facilitating stone access, reducing operative time, and lowering complication rates. Postoperatively, patients in the silodosin group also achieved a higher stone-free rate and required fewer analgesics compared to the placebo group. Conversely, a study by Sokhal et al. reported that alpha-blocker use before URS could be disadvantageous [17]. In a case-control study, patients with mid- and lower ureteric calculi received 0.4 mg of tamsulosin prior to URS. In this group, ureteroscope passage, stone access, and intraoperative maneuvering were technically more challenging than in the group without preoperative alpha-blocker therapy. One of the main challenges in URS is the difficulty of inserting the ureteroscope through the ureteral orifice, which may be unsuccessful due to its narrow diameter. This limitation is partly explained by the abundance of alpha receptors in the distal ureteric smooth muscle, which facilitate smooth muscle contraction.

This study has several limitations. First, it was conducted at a single center with a relatively small sample size of 88 patients, which may limit the generalizability of the findings. The short follow-up period of one week restricted the assessment of long-term outcomes, such as stone recurrence or delayed complications. Additionally, all procedures were performed by a single surgeon, which, although ensuring procedural consistency, may introduce operator bias and limit the applicability of results to broader clinical settings. Lastly, this study focused exclusively on distal ureteric stones, so the findings cannot be extrapolated to stones located in other parts of the ureter.

## Conclusions

Preoperative alpha-blocker therapy, particularly with silodosin, significantly improves the success rate and reduces complications during URS and lithotripsy for distal ureteral stones. The study demonstrated that patients receiving alpha-blocker therapy had a notably higher success rate (81.82%) compared to the placebo group (54.55%), a difference that was statistically significant. These findings support the use of alpha-blockers as an effective preoperative treatment to facilitate stone passage, enhance ureteral access, and reduce the need for ureteral dilatation during the procedure. While complications such as septicemia, bleeding, and UTIs occurred in both groups, their incidence was lower in the alpha-blocker group, suggesting a potential benefit in reducing postoperative complications. Although the differences in complication rates were not statistically significant, the trend toward fewer complications in the alpha-blocker group highlights its additional value in enhancing patient safety during and after the procedure.
